# A master of all trades – linking retinoids to different signalling pathways through the multi-purpose receptor STRA6

**DOI:** 10.1038/s41420-021-00754-z

**Published:** 2021-11-16

**Authors:** Vinesh Dhokia, Salvador Macip

**Affiliations:** 1 Mechanisms of Cancer and Aging Laboratory, Department of Molecular and Cell Biology, University of Leicester, Leicester, UK; 2 FoodLab, Faculty of Health Sciences, Universitat Oberta de Catalunya, Barcelona, Spain

**Keywords:** Targeted therapies, Senescence, Calcium signalling, Extracellular signalling molecules, Nuclear receptors

## Abstract

Retinoids are a group of vitamin A-related chemicals that are essential to chordate mammals. They regulate a number of basic processes, including embryogenesis and vision. From ingestion to metabolism and the subsequent cellular effects, retinoid levels are tightly regulated in the organism to prevent toxicity. One component of this network, the membrane receptor STRA6, has been shown to be essential in facilitating the cellular entry and exit of retinol. However, recent data suggests that STRA6 may not function merely as a retinoid transporter but also act as a complex signalling hub in its own right, being able to affect cell fate through the integration of retinoid signalling with other key pathways, such as those involving p53, JAK/STAT, Wnt/β catenin and calcium. This may open new therapeutic strategies in diseases like cancer, where these pathways are often compromised. Here, we look at the growing evidence regarding the novel roles of STRA6 beyond its well characterized classic functions.

## Facts

Retinoids have many key roles in different biological processes, ranging from embryogenesis and vison to immunity and reproduction, and they are also involved in diseases, such as cancer. They are essential for life and are obtained through the diet.

STRA6 is the membrane receptor the retinol-RBP complex and thus the cellular gateway for retinol signalling. Once inside the cells, retinoids trigger a series of events that lead to activation of nuclear receptors (RARs and RXRs) and transactivation of several target genes.

Recent findings have shown that STRA6 is, in fact, a central node of a hub that brings together different pathways, including p53, JAK/STAT, Wnt/β catenin and calcium signalling. Thus, STRA6 links retinoids with essential cellular responses though mechanisms not yet fully defined. This could have therapeutic implications.

## Open questions

How does STRA6 exert its retinoid-independent functions?

Are there alternative forms of STRA6 that are not membrane-bound?

Can we modulate cell fate decisions by chemically activating or inhibiting STRA6, and thus propose novel therapies for diseases such as cancer?

## Retinoids: a brief historical perspective

Retinoids are a group of natural and synthetic compounds derived from Vitamin A, which have been shown to have roles in embryogenesis, vison, immunity, reproduction and cell differentiation, among others [1]. Retinoids were defined in 1982 by the International Union of Pure and Applied Chemistry - International Union of Biochemistry (IUPAC-IUB) as consisting of four isoprene units joined together in head to tail manner, which may be derived from a monocyclic parent compound containing five carbon–carbon double bonds with a functional group at the terminus of acyclic group. Now, Vitamin A is a term often used to describe all retinoid derivatives, such as Retinol, Retinoic acid (RA) and all-*trans* retinoic acid (ATRA).

Vitamin A was known indirectly 3500 years ago by the ancient Egyptians, who applied fresh Ox liver to the eye to cure night blindness [2]. Throughout the 19th century and the advent of long sea voyages, the diaries and journals of ship doctors revealed the effects of nutrient-related diseases, of which lack of Vitamin A was common [3]. Since then, altered Vitamin A homeostasis has been observed in several conditions, such as cancer [4], Alzheimer’s [5] and skin disorders [6]. From the 1930’s, when T Moore first deduced that the source of vitamin A was β Carotene [7], through the 1960’s and the Nobel prize winning discovery of G Wald of 11-cis retinal as the chromophore in visual pigments [8], up to today’s use of retinoids in oncology, retinoid biochemistry remains of immense interest in numerous fields. In 1931 Paul Karrer, the swiss chemist, described the chemical structure of Vitamin A, for which he was awarded a Nobel prize in chemistry in 1937 [9, 10]. The mass production of Vitamin A was started in 1947 by Otto Isles at Hoffman-La Roche building, on the work of chemists Arens and Van Doep [11, 12].

**Fig. 1:**
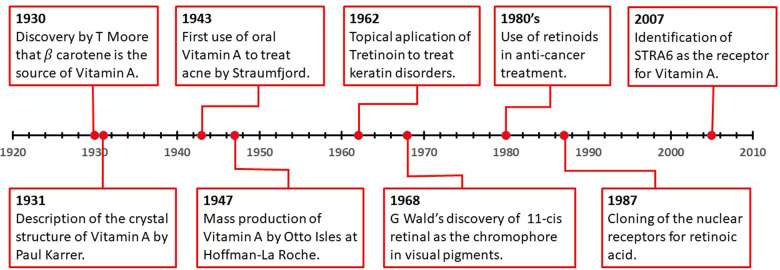
A timeline of key developments in retinoid research history. The last 100 years of retinoid biochemistry has led to the elucidation of many of its signaling components and the clinical applications of retinoids in dermatology and cancer.

The use of Retinoids in dermatology began in 1943, after Straumfjord gave oral Vitamin A to treat acne [13]. Topical application of Tretinoin, a metabolite of Vitamin A, was first used to treat various keratin disorders by Stüttgen in 1962, after which an explosion of various retinol-based therapies were used in potential anti-aging treatments [14]. The anti-cancer effects of retinoids began to be investigated through the 1970’s and 1980’s and remain controversial to this day [2]. From the 1980’s onwards, advances in biochemical and structural analyses allowed the elucidation of retinoid signalling within the cell, revealing the diverse biochemistry of the retinoid family. A timeline of key events in the history of retinoids can be seen in Fig. 1.

## Vitamin A metabolism

**Fig. 2:**
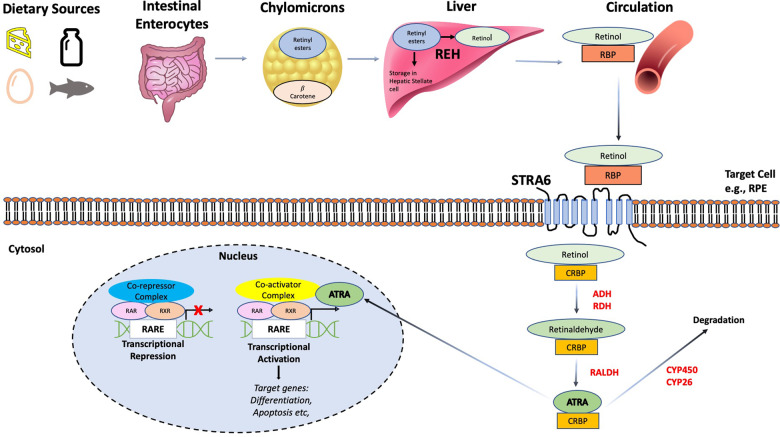
Overview of retinoid metabolism and signalling. Retinyl esters and β carotene obtained from dietary sources, such as carrots and fish, are absorbed in intestinal enterocytes and transported to the liver via chylomicron lipid transporters. Here, they may be stored in stellate cells or converted to retinol via retinol ester hydrolases (REH). Retinol is transported to target cells, such as retinol pigment epithelium cells (RPE), bound in the bloodstream to retinol binding protein (RBP). Cellular uptake of retinol is driven by the membrane receptor stimulated by retinoic acid 6 (STRA6). Once internalized, retinol is converted into retinaldehyde by alcohol dehydrogenase (ADH) or retinol dehydrogenase (RDH). Retinaldehyde is then converted into the biologically active form of retinoic acid or all-trans-retinoic acid (ATRA) by a family of retinaldehyde dehydrogenases. ATRA may either be degraded by a family of cytochrome p450/26 enzymes or transported to the nucleus by cellular retinol binding protein (CRBP), where it binds to RAR/RXR heterodimers bound to retinoic acid response elements (RAREs). In an inactive state, repressor complexes prevent transcription by blocking access of transcriptional components. Upon ligand binding, a conformational change occurs, allowing the recruitment of co-activator complexes that permit the transcription of genes under control of RAREs and may ultimately decide cell fates such as differentiation or apoptosis.

All chordates are dependent on Vitamin A for life, because this liposoluble factor cannot be synthesized de novo and must be taken from the diet. Vitamin A is obtained from dietary β-carotene from sources such as milk, eggs, fish liver oils and coloured fruits and vegetables such as carrots [15]. Once ingested, dietary carotenoids and retinyl esters are converted into retinol in the intestinal lumen by retinyl ester hydrolases and may enter lymph circulation in a chylomicron complex with phospholipids and triglycerides [16] (Fig. 2). Triacylglycerol hydrolysis occurs in lymph circulation, and the chylomicron remnants are removed by the hepatocytes in the liver [17]. There, retinyl esters are hydrolysed, and unesterified retinol may bind to retinol binding protein (RBP) and be secreted into plasma, resulting in a steady concentration in blood of 1–2 μM [15]. Around 70% of all retinoids in the body in the form of retinyl esters are stored in hepatic stellate cells in the liver [18]. In healthy individuals, 90% of the blood RBP is bound to retinol (which is then called holo-RBP) and 10% is unbound (called apo-RBP) [19]. Holo-RBP may also be bound to the serum protein Transthyretin (TTR), which prevents glomerular filtration and retinol degradation by the kidney, ensuring delivery to target tissues such as heart, muscle, lungs, reproductive organs, adipose tissue and bone marrow [20].

## The retinoid signalling pathway

Once inside the cells of target tissues, retinol binds to cellular retinol binding protein 1 (CRBP1) and is oxidized to retinaldehyde by retinol dehydrogenases (RDH) and alcohol dehydrogenases (ADH) [21] (Fig. 2). Retinaldehyde is then oxidised to RA by a family of three retinaldehyde dehydrogenases (RALDH1, RALDH2 and RALDH3) [22]. If RA is to be degraded, it is further oxidised by a family of three cytochrome p450 enzymes (Cyp26A1, Cyp29B1 and Cyp26C1) [4]. RA can then be delivered to the nucleus by cellular-RA-binding protein 2 (CRABP2), where it exerts its transcriptional activity via two families of nuclear receptors: retinoic acid receptors (RARs) and retinoid X receptors (RXRs) [23].

Both RARs and RXRs belong to the nuclear hormone receptor superfamily and function as ligand-activated transcription factors [24]. In 1987, the first RAR, later known as RARα, was cloned independently by the groups of P Chambon and R Evans [25, 26]. The RAR family has now grown to also include RARβ and RARγ. The sub-family of RXRs similarly consists of RXRα, RXRβ and RXRγ, though the sequences of RXRs differ markedly with that of RARs [27]. The RARs are activated by ATRA, 9-cis-RA and 13-cis-RA, whilst the only natural ligand for RXRs is 9-cis-RA [1]. The RAR/RXR heterodimer can bind to the retinoic acid response elements (RAREs) or retinoid X response elements (RXREs) of target genes and allow transcriptional activation. The canonical RARE sequence is a repeat of AGGTCA with either a two (DR2) or five (DR5) nucleotide spacer, whilst RXREs are commonly separated by a single nucleotide (DR1) [28]. In the absence of ligand, the RAR in the heterodimer recruits corepressors, which in turn recruit histone deacetylase complexes (HDACs). This results in condensed chromatin and DNA to be inaccessible to the transcription machinery [28]. Upon ligand binding, the RAR/RXR heterodimer may bind to co-activators such as the p160 subfamily of steroid receptor coactivators SRC-1 and SRC-3, which contain histone acetyltransferase that allows chromatin to open up and transcription to occur [29]. There are over 3000 human genes, including STRA6, associated with RAREs and involved in a wide variety of biological functions, which underlie the complexity and importance of retinoid signalling [30, 31].

## Retinoids in development

RA has long been known as a morphogen during embryogenesis, being particularly vital in the development of the trunk and eyes. Indeed, the role of vitamin A in eye development is already highlighted in a paper from the 1930’s, in which there is a description of a pregnant gilt on a vitamin A deficient diet that gave birth to 11 piglets without eyes [32]. Further vitamin A deficiency studies in chicken and mammalian embryos have revealed the vital role of RA in developing the lung, forebrain, hindbrain, skeleton, forelimb buds and heart [33]. The fact that RA levels are tightly controlled across the developing embryo creates distinct boundaries and gradients between populations of cells, allowing the differentiation of stem cells [34]. Exogenous application of ATRA to human or rodent embryos results in malformations of the anterior/posterior axis and the structures arising from it, including the neural tube, highlighting the teratogenic effects of retinoids [35].

The development of the eye is a result of paracrine signalling of RA across embryonic eye tissues by all three RALDHs, with specific expression patterns [36]. During optic cup formation, from E9.5 to E10.5 in mouse embryos, RA generated by RALDH2 in the perioptic mesenchyme and RA generated by RALDH3 in the retinal pigment epithelium (RPE) move toward the neural retina to allow invagination of the optic vesicle epithelium [36]. During anterior eye formation from E10.5-birth, RALDH3 expression stops in the RPE and is instead expressed in the ventral neural retina, whilst RALDH2 is also no longer expressed in the perioptic mesenchyme and RALDH1 is expressed in the dorsal neural retina [21].

Regulation of differentiation by RA lays in its ability to control gene expression, particularly the Homeobox or Hox genes, a family of transcription factors that bind to DNA through a helix-turn-helix motif called a homeobox or homeodomain [37]. In the presomitic mesoderm, RA is released and travels to the posterior foregut endoderm and posterior neuroectoderm to induce homeobox genes in the trunk and posterior hindbrain tissues [21]. In addition to activating genes, RA also represses expression of fibroblast growth factor 8 or Fgf8 in the caudal epiblast to allow body axis extension [38]. This repression relies on a RARE upstream of Fgf8 recruiting polycomb repressor complex 2 (PRC2) and nuclear receptor co-repressors NCOR1 and NCOR2 (SMRT), which promotes repressive chromatin. This provided the first in vivo evidence of direct RA gene repression through RARE [38, 39].

## Retinoids in cancer

Given the large number of genes associated with RAREs, RA may have both suppressive and promotional roles in cancer. Abnormal retinoid signalling occurs in numerous malignancies, with breast, kidney, prostate and skin cancers all having low intracellular retinyl ester levels [40]. A well-documented tumour suppressor is RARβ2, a RARβ splice variant. RARβ2 expression is lost early in many tumour types and can be epigenetically silenced in human cancers [41]. Restoration of RARβ2 expression in breast cancer can lead to growth inhibition and a combination treatment of 5-Aza-CdR, an inhibitor of methylation, and ATRA, can produce a synergistic antineoplastic effect on DLD-1 colon cancer cells [42, 43]. Contradictory data, however, shows that silencing of RARβ2 in non-small cell lung cancer (NSCLC) can result in increased apoptosis and reduced proliferation, highlighting the complexity and variability of retinoid signalling in neoplasia [44].

Retinoids have been used extensively in both the treatment and prevention of cancer, with varying degrees of success. One of the most successful uses is in the treatment of acute promyelocytic leukaemia (APL). In patients with APL, a reciprocal chromosomal translocation of the RARα gene on chromosome 17 and the promyelocytic leukaemia (PML) gene on chromosome 15 results in a PML-RARα fusion protein [45]. This fusion protein exhibits enhanced recruitment of co-repressor and HDAC complexes, resulting in silencing of RA responsive genes involved in differentiation and apoptosis [45]. Treatment with ATRA can induce terminal differentiation of leukemic promyelocytes into mature granulocytes, which results in significantly improved patient outcomes [46]. A combination treatment of ATRA and arsenic trioxide (As_2_O_3_) can increase overall survival and complete remission in these patients when compared to ATRA alone [47]. In other cancers, however, results have been mixed. In pancreatic cancer, a pilot phase II study of 13-cis-RA in combination with Gemcitabine saw no improvement in response rate despite being well tolerated [48]. On the other hand, children with high-risk neuroblastoma who received total-body irradiation, myeloablative chemotherapy or autologous bone marrow transplant followed by 13-cis-RA treatment demonstrated increased 3-year event-free survival when compared to those without 13-cis-RA [49].

This variability in responses of retinoids in cancer treatment highlights the heterogeneity of the altered retinoid pathway, not only between cancers but between individuals as well. The era of personalized medicine may mean stratifying patients into those with preserved RA-inducible genes that could enhance the response to chemo/radiotherapy when stimulated by retinoids. In addition, crosstalk or overlaps with the growing number of pathways associated with retinoid signalling may need to be considered to deliver truly personalized treatments that address the resistance or sensitivity of a particular cancer to retinoid-based combination therapies.

## STRA6 as the specific RBP receptor

Being both hydrophilic and lipophilic allows retinol to enter cells via diffusion through the cell membrane. However, the retinol-RBP complex mainly undergoes receptor mediated endocytosis via the stimulated by retinoic acid gene 6 (STRA6) [50]. The existence of a membrane receptor for RBP was proposed in 1975 by J Heller, who observed binding of iodinated bovine and human RBP to bovine pigment epithelium cells to an unknown receptor with high affinity [51]. However, the identity of this receptor remained unknown for the next thirty years. STRA6, a 75-kDa protein, was first identified as a RA responsive gene in mice, but it was not until later that it was found to be the elusive receptor for RBP [50, 52]. STRA6 fulfilled the criteria expected of an RBP binding protein, namely, uptake of vitamin A, conferring binding of RBP in transfected cells and cellular localization in tissues expected to have such a receptor [50]. Further structural analysis of STRA6 revealed four intracellular domains, five extracellular domains and nine transmembrane domains, whilst mutagenesis studies narrowed the RBP binding region between transmembrane domains VI and VII [53, 54].

## STRA6 in disease: retinoid dependent and independent effects

Mutations in STRA6 have been found to contribute to the inherited and fatal disease known as Mathew Wood syndrome (MWS). MWS was first described in two siblings with anophthalmia/microphthalmia and associated pulmonary defects [55]. In 2007, STRA6 mutations were identified in patients exhibiting MWS syndrome and a range of other malformations, including lung hypoplasia, mental retardation and congenital heart defects [56]. A total of 44 patients with bi-allelic STRA6 mutations have already been identified, displaying a diverse phenotype that bears no correlation to either the type or the position of the mutation [57]. Mutations can be found throughout STRA6, with nonsense and missense mutations resulting in a truncated protein contributing to MWS, suggesting that a full length STRA6 is required for it to function correctly [58, 59]. Nevertheless, a high concentration of mutations is located at the long intracellular C terminal tail, including T644M (in a SH2 domain) and R655C (a phosphorylation site), potentially indicating a key role for this part of STRA6 in its function [56]. Given the correlation with its developmental activity, these effects of the functional impairment of STRA6 are likely linked to its key upstream role in retinoid signalling.

However, STRA6 may also be implicated in disease independently of retinoids. Whilst various components of the retinoid pathway have been implicated in cancer, there is increasing evidence that STRA6 itself may have a role in tumorigenesis. In gastric cancer (GC), STRA6 has been found to be upregulated, and its knockdown inhibited Wnt/β catenin signalling [60]. Using bioinformatics to predict miRNA, Lin and colleagues found miR-873 negatively regulates STRA6 and has a tumour suppressive role in GC, which could be reversed by STRA6 overexpression [60]. This idea of controlling STRA6 expression by miRNAs opens up possible therapeutic intervention, given that miRNA dysregulation has been found in a number of cancers and could contribute to sustained proliferative signals [61].

STRA6 single nucleotide polymorphisms (SNPs) have also been associated with EGFR mutations in metastatic NSCLC patients [62]. In particular, two TT genotype SNP’s, rs4886578 and rs736118, positively correlated with a mutated EGFR and non-smoking history in NSCLC patients over 60 years of age, leading to the speculation that these STRA6 SNPs may serve as biomarkers in locally advanced and metastatic NSCLC patients. To date, there is no evidence of STRA6 interacting with the EGFR pathway. Further studies are necessary to determine if components of the EGFR pathway are targets of RAR genes or whether STRA6 itself may somehow be influencing EGFR signalling. In addition, both SNP’s from this study also positively correlated with type 2 diabetes mellitus (T2DM) in a study involving the south Indian population [63]. The rs736118 SNP is also associated with T2DM in the southern Han Chinese population [64]. This particular SNP is located in the C terminal tail of STRA6, and a G→A polymorphism that results in a methionine to isoleucine change could alter cell surface expression and trafficking [63].

These findings provide a glimpse of the role that STRA6 mutations and SNPs may play in disease development, though much work remains to be done, particularly in variations found in populations around the world and the exact mechanisms that underpin STRA6 contributions. Altered retinol homeostasis through aberrant STRA6 function and expression could potentially lead to an excess in proliferative signals, thus driving tumour development and other pathologies. However, it is also important to consider the disruption of the novel STRA6 functions that fall outside retinoid signalling when studying its role in disease.

## Novel functions of STRA6 (I): a bidirectional transporter and role in calcium signalling

When STRA6 was first identified, it represented both a new class of membrane receptor and a membrane transport protein, thus opening up the possibility of STRA6 having roles aside from vitamin A uptake. As target cells are exposed to a steady supply of vitamin A, a constant influx could lead to toxic levels accumulating within the cell. Therefore, it is reasonable to assume that a mechanism exists to maintain a tight intracellular vitamin A homeostasis. Consistent with this, STRA6 has been shown to function as a bidirectional transporter of vitamin A, and this mechanism is regulated by Calmodulin (CaM) [65]. STRA6-expressing NIH 3T3 cells preloaded with retinol have been shown to release more retinol into culture media than cells that do not express STRA6 [66]. Moreover, mouse embryonic STRA6 expression has been shown to be increased in response to excess dietary retinoids, and LRAT deficient mouse embryos, which theoretically should have higher intracellular retinol due to an inability to convert retinol to retinol ester, also display higher STRA6 levels [67]. In addition, applying pure extracellular apo-RBP to cells can cause retinol efflux in a STRA6-dependent manner [68]. When exposed to human serum containing both holo-RBP and apo-RBP, holo-RBP competes with apo-RBP for binding to STRA6 and is able to block Sta6 mediated retinol efflux, instead causing retinol influx. All this supports the idea that STRA6 may be upregulated to export excessive intracellular retinol.

The interaction of STRA6 with CaM first came to light using cryo-electron microscopy to resolve the zebrafish STRA6 structure, which found that CaM to be bound tightly to STRA6 [69]. This interaction involved a STRA6 dimer bound to CaM in a non-canonical arrangement, predominantly involving the cytoplasmic C terminal tail of STRA6 interacting with CaM and a large extracellular hydrophobic outer cleft that may be used for retinol release from RBP. This novel interaction was confirmed by purifying and identifying STRA6 interacting proteins from mammalian RPE cells using affinity purification and mass spectrometry [65]. Mutating a residue (R635A) in a predicted CaM site at the C terminal tail of STRA6 significantly reduced the binding, whilst higher intracellular calcium levels also strengthened the interaction. In addition, the higher intracellular calcium levels resulted in greater Vitamin A efflux through STRA6, and CaM enhanced the binding of STRA6 to apo-RBP compared to holo-RBP. These findings suggest that intracellular calcium levels may dictate vitamin A homeostasis through STRA6 and CaM. CaM has many downstream targets involved in processes as diverse as apoptosis, inflammation, muscle contraction and immunity [70], to which STRA6 may be contributing.

## Novel functions of STRA6 (II): involvement in signalling pathways

There is evidence of STRA6 being also an important component of other signalling networks, including the Wnt and the JAK/STAT pathways. Wnt is involved in embryonic development, cell migration, cell fate decisions and is hyperactivated in a high percentage of human cancers [71]. The canonical Wnt pathway results in translocation of β catenin from the cytosol to the nucleus upon binding of Wnt ligands to frizzled receptors at the plasma membrane, leading to gene transcription by complexing with TCF/LEF transcription factors [71]. STRA6 mRNA was found to be upregulated in mouse C57MG mammary epithelial cells when stimulated by Wnt-1 and Wnt-3A, and synergistically induced by RA and Wnt-1 [72]. Further studies, in which mice with breast tumours containing mutations in the Wnt-1/β catenin pathway were given oral vitamin A, also resulted in increased STRA6 expression [73]. This suggests that β catenin may enhance RAR dependent expression of target genes independently of TCF/LEF by recruiting coactivators or displacing corepressors. The exact mechanism of how the Wnt and retinoic acid pathways converge through STRA6 remains to be fully elucidated. The RARγ receptor maybe key in mediating the expression of STRA6 and RAR responsive genes [74]. In addition, β catenin has been shown to interact directly with RARs in a RA-dependent manner and potentiate the activity of RA on RAR responsive promoters [75]. This may ultimately lead to increased STRA6 expression, which may alter cell fate decisions particularly during embryogenesis, a process in which both STRA6 and Wnt signalling are heavily involved.

Another signalling cascade that STRA6 is involved in is the Janus kinase/signal transducers and activators of transcription (JAK/STAT) pathway. The JAK/STAT pathway in mammals is utilised by cytokines and growth factors to influence cell proliferation, differentiation, migration and is essential in numerous processes such as immune development, mammary gland development and sexually dimorphic growth [76]. Upon cytokine binding to its receptor, a conformational change in the cytoplasmic portion of the receptor allow activation of JAKs and phosphorylation of a tyrosine residue on the receptor [77]. This allows recruitment of STATs to the receptor, which are also phosphorylated by the JAKs and activated. This leads to dissociation from the receptor, dimerization and translocation to the nucleus, where binding to gamma activated site (GAS) enhancers can occur [77]. Retinol-RBP binding to STRA6 results in STRA6 phosphorylation, leading to activation of JAK2 and STAT5 [78]. Conversely, mutation of the tyrosine residue Y643 in the presumed SH2 domain of STRA6 resulted in diminished STRA6 phosphorylation. In addition, the phosphorylation of STRA6 on residue Y643 by JAK2 was found to be necessary for retinol transport. This interdependence of retinol uptake and phosphorylation of STRA6 is unique and has led to considering STRA6 a new class of “cytokine signalling transporter” [78]. Moreover, an increased ratio of apo/holo RBP4 concentration enhanced the JAK2/STAT5 cascade, resulting in activation of the AC6 catalysed cAMP/PKA/JNK1/p38 pathway and ultimately causing apoptosis via increased active caspase 3 in HK-2 and HUVEC cells [79]. An increase in STRA6 expression and a decrease in CRBP-1 and RARα expression was also observed, together with an increased apo/holo RBP4 concentration. The altered apo/holo RBP serum concentration may block RA uptake by STRA6, leading to altered intracellular signalling and resulting in apoptosis.

## Novel functions of STRA6 (III): involvement in stress responses

Functioning mainly as a transcription factor, p53 is activated after a variety of stresses, leading to cell fate responses such as cell cycle arrest, DNA repair or apoptosis, and thus it is often referred as the ‘guardian of the genome’ [80]. It has long been known that RA stimulates the transactivation of p53 in a retinoid receptor dependent manner, but independent of p53 stability or DNA binding [74]. Direct evidence of ATRA upregulating p53 levels were observed by increased p53 protein and mRNA levels and the apoptotic caspases-3, -6, -7 and -9 after the addition of ATRA to keratinocytes (KC). This resulted in apoptosis when DNA damage was applied to these KC, but not in ATRA-deficient KC. Of note, spontaneous apoptosis did not occur when ATRA was added, but only after DNA damage was applied, suggesting an enhancement of p53 apoptotic activities by the retinoid pathway that mediated an ATRA-dependent sensitization of KC to apoptosis.

Indeed, it was later discovered that STRA6 sits at the centre of a crosstalk network between the p53 and RA pathways [81]. STRA6 was found to be a p53 responsive gene and was thus induced in response to genotoxic stress in the form of UV radiation, doxorubicin or oxidants. Transfection of STRA6 into cancer cells was able to turn an initial arrest response into apoptosis, indicating that STRA6 can affect p53 cell fate decisions by modulating its activity. STRA6 was also found to increase intracellular ROS and mitochondrial depolarization, comparable to the levels induced damage responses. Consistently, a decrease in oxidative damage to DNA after stress was observed when STRA6 was knocked down. Of note, these experiments were done in RA-resistant HCT116 cells, which indicates that the role of STRA6 in the p53 is likely independent of downstream RA signalling.

This evidence suggests that STRA6 may take part in stress responses by contributing to the intrinsic apoptotic pathway through ROS release from the mitochondria. As both a p53- and RA-responsive gene, STRA6 may thus be involved in a positive feedback loop to enhance stress responses: RA induces STRA6 expression, which in turn increases p53 activity and leads to further STRA6 upregulation. This may be particularly effective in cells more sensitive to oxidative stress, and opens up a new angle when using Vitamin A metabolites in cancer therapy. The link between STRA8 and the p53 pathway could also help explain why the response of cancer cells to RA is so heterogeneous.

## Conclusions and challenges: STRA6– more than meets the eye

**Fig. 3:**
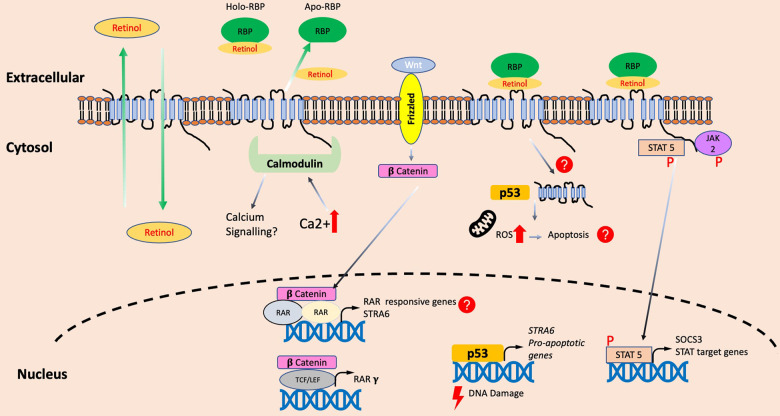
The multiple roles of STRA6. As well as a bi-directional transporter of retinol, STRA6 may bind to signalling molecules such as calmodulin to integrate calcium signalling with retinol transport. In addition, evidence has shown STRA6 to converge with various signalling pathways such as WNT, p53 and JAK/STAT to potentially determine cell fate decisions.

Evidence is now mounting that STRA6 is not only a transporter of vitamin A, but a key component of a growing array of cellular pathways (Fig. 3). Thus, STRA6 could be acting as a master switch in cell fate decisions, modulating key signalling pathways to trigger the adequate response. Retinoid and calcium metabolism, proto-oncogenic pathways (such as Wnt and JAK/STAT) and tumour suppressor mechanisms (p53) all seem to converge in this complex protein, which has the power to enhance or dampen signals to decide whether the cell survives or dies, despite not having itself the ability to significantly trigger pro-survival or pro-death signals.

These findings raise questions as to how the structure and location of STRA6 relate to these novel functions. The C terminus of STRA6 in particular warrants further attention, as it appears to be both a target for signalling cascades and required for retinol uptake. Given the number of mutations found in this region, a better understanding of how this C terminal tail relates to the whole receptor could aid therapeutic developments in disease states. Moreover, we still need to understand how a plasma membrane receptor can modulate responses that mostly take place in the cytosol and/or the nucleus, such as those elicited by the p53 pathway. Components of the apoptotic pathway could target and modify STRA6 into a form more suited to its apoptotic role. Consistent with this hypothesis, it has been found that STRA6 may take on a cytoplasmic localization after damage [81], which challenges the dogma of STRA6 being a fixed transmembrane protein. Whether this is a cleaved version of STRA6 or an alternative isoform still needs to be elucidated.

All this knowledge could eventually lead to translational applications. For instance, the fact that STRA6 tightly regulates vitamin A homeostasis whilst integrating signalling pathways to affect cell fate decisions may be particularly important in cancer therapy, where STRA6 may be a target to enhance retinoid combination therapy. Stratification of patients depending on their STRA6 expression and sensitivity to RA could help identify those cancers in which RA could be successfully used as adjuvants of chemo/radiotherapy. Given the diverse outcomes of the p53/Wnt-1/JAK/STAT pathways and their relevance in many malignancies, the options could be many.

Over time, our view of STRA6 has changed from being just a receptor to the potential orchestrator of a vital signalling cascade hub. This places STRA6 in a special new category of transmembrane proteins that needs to be further investigated. The field of STRA6 research is still in its infancy, having started <15 years ago, and it is likely that many more surprises still await. Future work on how STRA6 modulates all these different responses will be required to fully understand the true relevance of this complex protein.
